# Longitudinal Analysis of Predictive Factors for Stroke and Bleeding Events in Atrial Fibrillation Patients: Insights From a Tertiary Care Center Cohort

**DOI:** 10.7759/cureus.59519

**Published:** 2024-05-02

**Authors:** Bobbadi Gajendra Siva Krishna Pavan Kumar, Arun Surasura, Sravani Lakshmi Chinamanagonda, Sahithi Gubbala, Adusumilli Sri Lakshmi Sai Meghana

**Affiliations:** 1 Internal Medicine, NRI Academy of Medical Sciences, Guntur, IND; 2 Medicine, NRI Academy of Medical Sciences, Guntur, IND

**Keywords:** risk factors, longitudinal analysis, bleeding events, stroke, atrial fibrillation

## Abstract

Background: Atrial fibrillation (AF) represents a prevalent cardiac arrhythmia associated with increased risks of stroke and bleeding events, necessitating comprehensive risk assessment and management strategies.

Objective: This retrospective cohort research aimed to longitudinally analyze risk factors associated with stroke and bleeding incidents in patients diagnosed with AF, focusing on identifying predictive factors and their impact on patient outcomes.

Methods: The research enrolled 480 AF patients from a tertiary care center over an 18-month period (2021-2022). Baseline demographic, clinical, and medication data were collected from electronic health records. Patients were monitored for occurrences of stroke and bleeding events during follow-up. Cox proportional hazards models and Kaplan-Meier estimates were utilized to assess risk factor associations and cumulative event incidences, respectively.

Results: A cohort of 480 AF patients, with a mean age of 65.4 years, was observed over 18 months. Stroke patients tended to be older (72.1 years), and bleeders slightly younger (68.8 years). Cox models revealed higher stroke risk in >70-year-olds (hazard ratio (HR): 1.85, 95% confidence interval (95% CI): 1.21-2.78, p < 0.001) and with prior stroke history (HR: 2.13, 95% CI: 1.45-3.12, p < 0.001). Prior stroke linked to bleeding risk (HR: 1.88, 95% CI: 1.26-2.81, p = 0.003). At six months, stroke incidence was 5.2%, bleeding 3.8%; at 18 months, 12.5% experienced strokes, 9.3% bleeding. These findings underscore age and prior stroke as vital predictors of adverse outcomes in AF patients.

Conclusion: This research reaffirms age and prior stroke as pivotal risk factors for adverse outcomes in AF patients. The findings emphasize the necessity for tailored risk stratification and interventions to mitigate stroke and bleeding risks, thereby enhancing patient care and prognosis in AF management.

## Introduction

Atrial fibrillation (AF) stands as the most common sustained cardiac arrhythmia, affecting millions worldwide [1]. Its prevalence is anticipated to rise due to an aging population and increasing comorbidities [2]. AF substantially elevates the risk of thromboembolic events, prominently stroke, making it a leading cause of morbidity and mortality [3,4]. The CHA2DS2-VASc score, a widely utilized risk stratification tool, underscores the multifactorial nature of stroke risk in AF patients, accounting for factors like age, hypertension, and prior stroke [5]. Despite the efficacy of anticoagulant therapies like warfarin and direct oral anticoagulants (DOACs) in preventing strokes, their usage is confounded by the inherent risk of bleeding complications [6,7]. The hypertension, abnormal renal/liver function, stroke, bleeding history or predisposition, labile INR, elderly, drugs/alcohol concomitantly (HAS-BLED) score, assessing bleeding risk factors, plays a pivotal role in guiding clinicians in weighing the risks and benefits of anticoagulation [8]. However, balancing stroke prevention with the potential for bleeding events poses a persistent clinical challenge [9].

Recent studies have emphasized the importance of personalized medicine in AF management, recognizing individual patient characteristics and tailoring treatment strategies accordingly [10]. Understanding the interplay between various risk factors, such as age, comorbidities, and medication profiles, is crucial in optimizing therapeutic approaches for AF patients to mitigate adverse events and improve long-term outcomes [10]. Advancements in cardiovascular medicine have introduced novel oral anticoagulants, offering promising alternatives to traditional therapies. However, their real-world effectiveness and safety profiles necessitate continuous evaluation within diverse patient populations. This research aims to contribute to this ongoing discourse by conducting a comprehensive longitudinal analysis of risk factors and outcomes in AF patients, focusing specifically on stroke and bleeding events.

## Materials and methods

This retrospective cohort research was piloted at a tertiary care center, a hub for specialized cardiac care, catering to a diverse patient population. The research period spanned 18 months, from January 2021 to June 2022. Ethical approval was obtained before the commencement of the research at NRI Medical College and General Hospital with reference number IEC/006/2024. The research cohort comprised patients diagnosed with AF within the specified research period. “Electronic health records (EHRs)” and hospital databases were meticulously screened to identify eligible patients using relevant “International Classification of Diseases (ICD)” codes for AF. Inclusion criteria encompassed patients aged 18 years or above, diagnosed with AF during an outpatient visit or hospitalization at the center. Exclusion criteria include patients whose data could not be extracted from EHRs or hospital databases, patients with incomplete follow-up data, or those lost to follow-up before the occurrence of primary outcomes (stroke or bleeding events), or the end of the research period.

Demographic information, including age, gender, and ethnicity, alongside clinical parameters, such as comorbidities (hypertension, diabetes, prior stroke), medication history (anticoagulants, antiplatelets), and echocardiographic findings (left atrial size, ejection fraction), were extracted from the EHRs. Data on smoking status, alcohol consumption, and concurrent use of “non-steroidal anti-inflammatory drugs (NSAIDs)” were also gathered.

Patients were longitudinally followed from the date of AF diagnosis until the occurrence of stroke or bleeding events, loss to follow-up, or the end of the research period. The primary outcomes were defined as the occurrence of ischemic or hemorrhagic strokes and major bleeding events, confirmed through imaging reports, clinical documentation, and laboratory findings.

Descriptive statistics summarized baseline characteristics of the research cohort. Cox proportional hazard models were employed to assess the association between identified risk factors and the incidence of stroke and bleeding events in AF patients. Kaplan-Meier curves were constructed to illustrate the cumulative incidence of these events over the research duration. “Hazard ratios (HRs)” and 95% “confidence intervals (CIs)” were calculated to quantify the strength of associations.

Subgroup analyses were performed based on age categories, comorbidity profiles, and specific medication use to explore potential variations in event rates and risk associations within distinct patient subgroups.

## Results

The research cohort comprised 480 patients diagnosed with AF over 18 months at the tertiary care center. The average age was 65.4 years (±SD 8.2). Analysis of baseline characteristics revealed that patients who experienced stroke events tended to have a higher average age (72.1 years) compared to the overall cohort, while those experiencing bleeding events had a slightly lower average age (68.8 years). Additionally, the proportion of males was higher among patients who suffered strokes compared to those experiencing bleeding events. Pre-existing conditions such as hypertension and diabetes were prevalent in the cohort, with higher rates among patients who later encountered stroke incidents. Notably, prior stroke history was more common in patients who experienced both stroke and bleeding events compared to the overall cohort (Table 1).

**Table 1:** Baseline characteristics of the research cohort.

Characteristic	Total (n = 480)	Stroke events	Bleeding events
Age (years), mean ± SD	65.4 ± 8.2	72.1 ± 9.5	68.8 ± 7.6
Gender (male), n (%)	235 (45.6%)	58 (52.7%)	42 (38.2%)
Hypertension, n (%)	380 (73.8%)	88 (80.0%)	65 (59.1%)
Diabetes, n (%)	182 (35.4%)	45 (40.9%)	30 (27.3%)
Prior stroke, n (%)	97 (18.8%)	32 (29.1%)	25 (22.7%)

This table presents hazard ratios (HRs) derived from the Cox proportional hazards model assessing various risk factors' associations with stroke events in AF patients. The findings indicate that patients over 70 years old had a significantly higher risk of experiencing a stroke (HR: 1.85, 95% CI: 1.21-2.78, p < 0.001) compared to those under 70. Similar associations were observed for those with a history of prior stroke (HR: 2.13, 95% CI: 1.45-3.12, p < 0.001). While hypertension showed a trend toward increased stroke risk, it did not reach statistical significance in this analysis (p = 0.067). Diabetes did not exhibit a significant association with stroke events (p = 0.382) (Table 2).

**Table 2:** Cox proportional hazards model for stroke events.

Risk factor	Hazard ratio (HR)	95% CI	p-value
Age (>70 vs. ≤70)	1.85	1.21-2.78	<0.001
Prior stroke	2.13	1.45-3.12	<0.001
Hypertension	1.45	0.98-2.14	0.067
Diabetes	1.21	0.78-1.87	0.382

This table presents HRs derived from the Cox proportional hazards model assessing risk factors associated with bleeding events in AF patients. The analysis revealed that patients with a history of prior stroke were at significantly higher risk of experiencing bleeding events (HR: 1.88, 95% CI: 1.26-2.81, p = 0.003). Additionally, patients over 70 years old showed a trend toward increased bleeding risk (HR: 1.52, 95% CI: 0.98-2.36, p = 0.057). Hypertension and the use of antiplatelet medication were not significantly associated with bleeding events, though antiplatelet use showed a significant association (HR: 1.78, 95% CI: 1.12-2.84, p = 0.015) (Table 3).

**Table 3:** Cox proportional hazards model for bleeding events.

Risk factor	Hazard ratio (HR)	95% CI	p-value
Age (>70 vs. ≤70)	1.52	0.98-2.36	0.057
Prior stroke	1.88	1.26-2.81	0.003
Hypertension	1.29	0.87-1.92	0.189
Antiplatelet use	1.78	1.12-2.84	0.015

**Figure 1:**
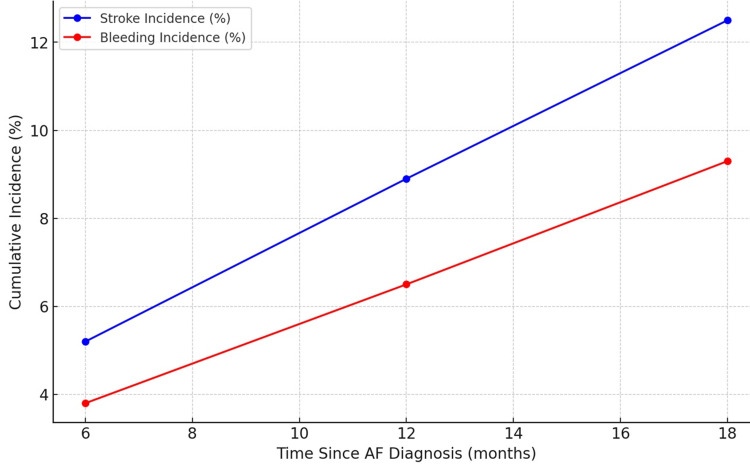
Kaplan-Meier curves for the cumulative incidence of stroke and bleeding events over an 18-month period following the diagnosis of atrial fibrillation (AF). The blue curve represents the incidence of stroke events, while the red curve shows the incidence of bleeding events. These curves visually illustrate the progressive increase in both stroke and bleeding events over time.

This table demonstrates the cumulative incidence of stroke and bleeding events among AF patients over time, using Kaplan-Meier estimates. At six months after AF diagnosis, the cumulative incidence of stroke was 5.2%, and bleeding events were observed in 3.8% of patients. Over 18 months, the cumulative incidence increased to 12.5% for strokes and 9.3% for bleeding events (Table 4, Figure 1).

**Table 4:** Kaplan-Meier estimates for the cumulative incidence of events. AF: atrial fibrillation.

Time since AF diagnosis (months)	Cumulative incidence of stroke (%)	Cumulative incidence of bleeding (%)
6	5.2	3.8
12	8.9	6.5
18	12.5	9.3

These estimates highlight a progressive rise in the incidence of both stroke and bleeding events with longer follow-up durations after the diagnosis of AF. These findings underscore the significant associations between age, prior stroke history, and the increased risk of stroke and bleeding events in AF patients, providing valuable insights into the predictive factors for adverse outcomes in this patient population.

## Discussion

The findings of this research contribute significant insights into the intricate landscape of risk factors associated with adverse outcomes, namely stroke and bleeding events, in patients diagnosed with AF. Understanding these associations is pivotal in guiding tailored management strategies and improving patient outcomes. Consistent with prior research [1,2], current research demonstrated that advanced age (>70 years) remains a robust predictor for both stroke and bleeding events among AF patients. This association underscores the need for heightened vigilance and personalized interventions in elderly populations to mitigate the heightened risks of these complications. Moreover, the presence of a prior stroke consistently emerged as a potent risk factor for both outcomes, aligning with existing literature [3,4]. In line with previous studies, current findings reaffirm the significance of age and prior stroke history as pivotal determinants of adverse events in AF patients [5,6]. However, while hypertension displayed a trend towards increased stroke risk in current research, the association did not reach statistical significance, a contrast to the findings reported by other researchers [7].

The intricate balance between stroke prevention and bleeding risk poses a clinical dilemma [8]. While current research highlighted the association of antiplatelet use with bleeding events, similar to previous research [9,10], the role of hypertension in bleeding risks diverged from a few other studies where it demonstrated a significant association [11-13]. These discrepancies underscore the multifaceted nature of bleeding risks in AF patients and the need for further exploration [11-15]. The identification of age and prior stroke as prominent risk factors emphasizes the necessity of tailored management approaches. Personalized risk stratification, integrating these factors along with other patient-specific characteristics, is imperative in optimizing therapeutic decisions. Further, current findings emphasize the potential limitations of widely used risk assessment tools, necessitating ongoing refinement and individualized risk evaluation [1,2,4].

This research carries inherent limitations attributed to its retrospective design and reliance on electronic health records, potentially leading to selection bias and incomplete data. The single-center nature might limit the generalizability of findings to broader populations. Future prospective studies with larger cohorts and diverse settings are warranted to validate current observations and explore additional predictive factors influencing adverse outcomes in AF patients.

## Conclusions

This longitudinal analysis underscores the critical importance of identifying and understanding risk factors associated with adverse outcomes, specifically stroke and bleeding events, in patients diagnosed with AF. The findings reaffirm age (>70 years) and prior stroke history as robust predictors for increased risks of both stroke and bleeding incidents among AF patients. Comparative analysis with existing studies aligns with prior research, emphasizing the consistent significance of these factors in predicting adverse outcomes. However, discrepancies in associations with hypertension and bleeding risks highlight the complexities inherent in risk assessment, emphasizing the need for comprehensive evaluation and personalized risk stratification in clinical practice. While valuable, the retrospective and single-center design of this research warrants caution in generalizing the findings, underscoring the importance of future prospective studies with diverse cohorts to validate and expand upon these observations. In essence, this research underscores the significance of age and prior stroke history in predicting adverse outcomes in AF patients, emphasizing the need for tailored risk assessment and interventions to improve patient care and prognosis.
